# BDNF rs6265 polymorphism methylation in Multiple Sclerosis: A possible marker of disease progression

**DOI:** 10.1371/journal.pone.0206140

**Published:** 2018-10-23

**Authors:** Viviana Nociti, Massimo Santoro, Davide Quaranta, Francesco Antonio Losavio, Chiara De Fino, Rocco Giordano, Nicole Palomba, Paolo Maria Rossini, Franca Rosa Guerini, Mario Clerici, Domenico Caputo, Massimiliano Mirabella

**Affiliations:** 1 Institute of Neurology, Fondazione Policlinico Universitario A. Gemelli IRCCS—Università Cattolica del Sacro Cuore, Rome, Italy; 2 IRCCS Fondazione Don Carlo Gnocchi, Milan, Italy; 3 Department of Physiopathology and Transplantation, University of Milan, Milan, Italy; Universita degli Studi di Napoli Federico II, ITALY

## Abstract

**Introduction:**

Brain-Derived Neurotrophic Factor (BDNF) and its most common polymorphism Val66Met are known to have a role in Multiple Sclerosis (MS) pathogenesis. Evidence is accumulating that there is an involvement of DNA methylation in the regulation of BDNF expression. The aim of this study was to assess in blood samples of MS patients the correlation between the methylation status of the CpG site near BDNF-Val66Met polymorphism and the severity of the disease.

**Methods:**

We recruited 209 MS patients that were genotyped for the BDNF Val66Met polymorphism. For each patient we quantitatively measured the methylation level of cytosine included in the exonic CpG site that can be created or abolished by the Val66Met BDNF polymorphism. Furthermore, we analyzed the clinical history of each patient and determined the time elapsed since the onset of the disease and an EDSS score of 6.0.

**Results:**

The genetic analysis identified 122 (58.4%) subjects carrying the Val/Val genotype, 81 (38.8%) with Val/Met genotype, and 6 (2.8%) carrying the Met/Met genotype. When the endpoint of an EDSS score of 6 was taken into account by means of a survival analysis, 52 failures (i.e., reaching an EDSS score of 6) were reported. When the sample was stratified according to the percentage of the BDNF methylation, subjects falling below the median (median methylation = 81%) were at higher risk of failure (IRD = 0.016; 95%CI = 0.0050–0.0279; p = 0.004).

**Conclusions:**

In patients with a high disease progression the hypomethylation of the BDNF gene could increase the secretion of the protective neurotrophin, so epigenetic modifications could be the organism response to limit a brain functional reserve loss. Our study suggests that the percentage of methylation of the BDNF gene could be used as a prognostic factor for disease progression toward a high disability in MS patient.

## Introduction

Multiple Sclerosis (MS) is an autoimmune, inflammatory, demyelinating, neurodegenerative disorder of the central nervous system (CNS) [1,2] in which CD4 and CD8 T-cells and B-cells action leads to a damage of the myelin [3,4]. Numerous scientific studies support the hypothesis of a role of the neurotrophic growth factors in the process of myelin repair [5,6]. As a member of the neurotrophin family, Brain-Derived Neurotrophic Factor (BDNF) has a role in the regulation of synaptic plasticity and in the neuronal differentiation and survival [7–10]. It also helps keeping the integrity of myelin [11,12]. The concentration of serum BDNF in stable relapsing-remitting (RR) MS patients is lower if compared with healthy individuals [13] but a higher one has been detected in patients during MS attacks [14]. Some studies provide evidence for a contribution of the BNDF in the process of remyelination of MS lesions [15]. In the human BDNF gene, a single-nucleotide polymorphism (SNP) rs6265 leads to an amino acid change from valine (Val) to methionine (Met) in position 66 (Val66Met) [16–18]. In MS, this polymorphism has been correlated with cognitive performance and measures of brain atrophy, with ambiguous results [19–21]. Accumulating evidence suggests the involvement of DNA methylation in the regulation of BDNF expression and in the pathology of several neurological diseases [22–25]. To date the methylation level of BDNF has not been investigated in the pathophysiology of MS. The aim of this study is to assess the correlation between the methylation status of CpG site of the BDNF gene and the progression of disability in MS patients.

## Methods

### Characteristics of the sample

The study sample was composed by 209 subjects (130 women; 62%), with mean age of 45.9 years (standard deviation [DS] = 12.7; median = 45). At the time of observation, 126 (60.3%) of the subjects were affected by RR MS, and 83 (39.7%) were affected by secondary progressive (SP) MS.

The mean duration of follow-up (equivalent to illness duration) was of 13.4 years (SD = 8.2; median = 12 years; maximum = 37 years); mean age at onset was 32.5 years (SD = 11.6; median = 32). Mean Annualized Relapse Rate (ARR) and MRI-ARR were respectively 0.54 (SD = 0.486) and 0.34 (SD = 0.290). The patients were enrolled between 2013 and 2014 and were followed at the Multiple Sclerosis Centre of Policlinico “A. Gemelli” in Rome and at the Multiple Sclerosis Unit at the Don Gnocchi Foundation in Milan. The local Ethical committee “Comitato Etico del Policlinico Gemelli” on December 2012 approved the study, and all patients gave their written consent to participate.

### DNA extraction

Genomic DNA was extracted from peripheral blood leukocytes using “salting-out” modified method and quantified by the Qubit 2.0 Fluorometer (Invitrogen, Carlsbad, CA, USA) according to the manufacturer’s instructions [26].

### High Resolution Melting (HRM) for BDNF rs6265 polymorphism genotyping

We analyzed the *BDNF* rs6265 polymorphism using HRM technique that characterizes nucleic acid samples based on their disassociation (melting) behavior using StepOnePlus thermocycler (Applied Biosystems, Foster City, CA, USA). The amplification of *BDNF* rs6265 polymorphism was carried out as previously reported [27]: in brief, the reaction mix (total volume of 20μl), contained 20 nanograms (ng) of genomic DNA, 0.5 μM of either forward/reverse primers and 1X of MeltDoctor HRM Master Mix (Applied Biosystems, Foster City, CA, USA).

Thermal cycling consisted of enzyme activation of 10 minutes at 95°C, followed by 40 cycles of each PCR step: (denaturation) 95°C for 15 seconds and (annealing/extension) 60°C for 1 minutes. Finally, melt curve/dissociation: denaturation at 95°C for 10 seconds, annealing at 60°C for 1 minutes, high resolution melting at 95°C for 15 seconds and annealing at 60°C for 1 minutes.

DNA melting curves of each samples were analyzed using high resolution melting software 3.1 (Applied Biosystems, Foster City, CA, USA) to identify homozygous or heterozygous genotypes. Each sample was assessed in triplicate.

### Sequencing

The genotypes showing different HRM profiles were sequenced using an ABI310 automatic sequencer and BigDye XTerminator Purification Kit (Applied Biosystems, Foster City, CA, USA). Specific primers (BD-SF: 5’-CCTACCCAGGTGTGCGGACC-3’; BD-SR: 5’-GTTTTCTTCATTGGGCCGAAC-3’) were designed to amplify an expected product size of 152 nucleotides and these fragments were bidirectionally sequenced to eliminate sequencing errors. PCR products were purified using SureClean PCR Purification Kit (Bioline, London, UK) following the protocol provided by the manufacturer.

### Bisulfite conversion

Bisulfite treatment of DNA converts all unmethylated cytosines to uracil, leaving methylated cytosines unaltered. Genomic DNA (2 μg in 20 μl) was treated with EZ DNA Methylation-Gold Kit (Zymo Research, Irvine, CA, USA) according to the manufacturer’s instructions. After bisulfite conversion, the genomic DNA was quantified by the Qubit 2.0 Fluorometer (Invitrogen, Carlsbad, CA, USA) according to the manufacturer’s instructions.

### DNA-methylation analysis of rs6265 BDNF by quantitative real-time PCR (qRT-PCR)

**Fig 1:**
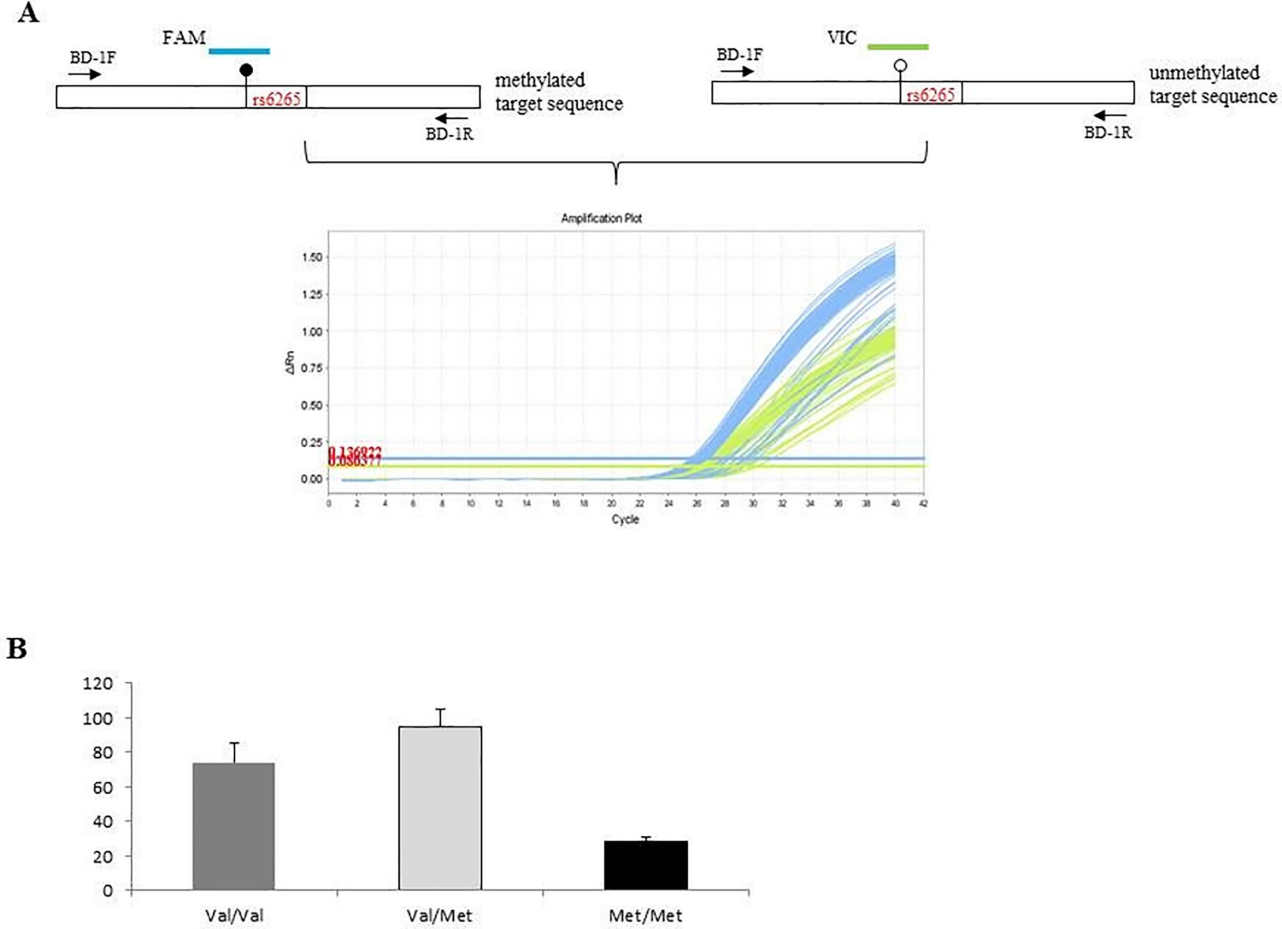
BDNF Methylation level determination and results. A) *Up-panel*, scheme of human *BDNF* exonic region that containing rs6265 SNP. The box indicates the coding region, black dot is CpG site with methylation presence and white dot CpG site devoid of methylation. The arrows indicates primers (BD-1F and BD-1R) used in this assay; lower panel, amplification plot representative of the amount of fluorescence dyes FAM (blue) and VIC (green) released during PCR that is directly proportional to the amount of PCR product generated from the methylated or unmethylated allele. B) Average percentage of methylation in MS patients carrying the Val/Val (gray column), Val/Met (light gray column) and Met/Met (black column).

In order to quantitatively measure the methylation level of cytosine included in the exonic CpG site that can be created or abolished by rs6265 *BDNF* polymorphism, we used bisulfite treatment of DNA and subsequent qRT-PCR. PCR primers are designed to amplify the bisulfite-converted region of 88 bp around *BDNF* rs6265 polymorphism. The binding sites of these primers lack CpG dinucleotides and, therefore, the nucleotide sequences in methylated and unmethylated DNA are identical after bisulfite treatment. Consequently, it is possible to amplify both alleles in the same reaction tube with one primers pair. Methylation discrimination occurs during probe hybridization by the use of two differently labeled internal TaqMan probes (FAM methyled and VIC unmethyled) (Fig 1A). qPCR was performed using a 96-well optical tray at a final reaction volume of 20 μl. Samples contained 10 μl of TaqMan Universal PCR Master Mix II, No AmpErase UNG (uracil-N-glycosylase) (Applied Biosystems, Foster City, CA, USA), 2 μl of bisulfite-treated DNA, 0.4μM each of the primers BD-1F 5’-GGTTTAAGAGGTTTGATATTATTGGTTGA-3’, BD-1R 5’-CTTCATTAAACCGAACTTTATAATCCTCAT-3’ and 0.2μM each of the fluorescently labeled probes [FAM- 5’ ATACGTGATAGAAGAGTTGTTG 3’(signal methylated), VIC- 5’ ATATGTGATAGAAGAGTTGTTG 3’ (signal unmethylated)] (Fig 1A). Initial denaturation at 95°C for 10 min to activate the AmpliTaq Gold DNA polymerase was followed by 40 cycles of denaturation at 95°C for 15 s and annealing and extension at 60°C for 1 min (StepOnePlus thermocycler, Applied Biosystems, Foster City, CA, USA). Primer and probe sequences were selected with the Primer Express software (Applied Biosystems, Foster City, CA, USA) following the guidelines of Wojdacz et al. [28].

PCR primers were designed to amplify the bisulfite-converted region of 88 bp around *BDNF* rs6265 polymorphism. The amount of FAM and VIC fluorescence released in each tube was measured as a function of the PCR cycle number at the end of each. The cycle number at which the fluorescence signal crosses a detection threshold is referred to as CT and the difference of both CT values within a sample (ΔCT) is calculated (ΔCT = CT_FAM_ − CT_VIC_). The percentage of methylation was calculated by taking the threshold cycles determined with each of both dyes:

Signal methylated: CT_(CG)_ (FAM)–represents the threshold cycle of the CG reporter (FAM channel).

Signal unmethylated: CT_(TG)_ (VIC)–represents the threshold cycle of the TG reporter (VIC channel). Percentage of methylation: C_meth_ = 100/[1+2^(Ct^_CG_^-Ct^_TG_)]% [29,30].

### Statistics

Mean comparisons were performed by using t tests with Levene’s test for equality of variance and ANOVA as requested. Frequency comparisons were performed by means of χ2 test with Fischer exact test when required.

The course of the disease was assessed by means of a survival analysis in which the outcome event was defined as the reaching of an Expanded Disability Status Scale (EDSS) score of 6 [31]. EDSS is the most used MS disability scale. It ranges between 0.0 (no abnormal neurological signs) to 10 (death for MS). An EDSS score of 6.0 defines the need of assistance for walking and is considered as a marker of unalterable disability progression [32]. The study design was retrospective, thus data about the clinical course of the disease were collected from clinical recordings of the enrolled subjects. Only subjects who were followed-up by the study centers since the onset of the disease were taken into account. The effect of the predictive variables was assessed by determining incidence rates (IRs) and incidence rates differences (IRDs) among groups of exposed and unexposed subjects. IRs were computed by means of person-times analysis, that allows to assess the incidence of the event of interest (outcome) in respect of time of exposition This kind of analysis is useful when retrospective data are taken into account, with different times of observation. Thereafter, a multiple variables Cox’s proportional hazard regression analysis was conducted, in which the following variables were entered: age; gender; clinical relapses; MRI relapses; ARR; annualized MRI relapse rate (MRI-ARR); treatment; rs6265 *BDNF* polymorphism; *BDNF* methylation; furthermore, given the reported interaction of genotype and methylation [25] an interaction term (polymorphism x methylation) was added to the model. Age, clinical relapses, MRI relapses and treatment were treated as variables changing over time; in other words, the values assumed by each of them was considered for each year of observation. Treatment was stratified as follows: IFN-β1a, IFN-β1b and Glatiramer Acetate were categorized as first line therapies; fingolimod and natalizumab as second line therapies; immunosuppressants (mitoxantrone, cyclophosphamide, azathioprine) as third line therapies. Gender, *BDNF* polymorphism, percentage of methylation of the *BDNF* gene, ARR and MRI-ARR were treated as variables not changing over time. Finally, in order to allow the comparison among groups of exposed and not exposed subjects, the percentage of *BDNF* gene methylation was dichotomized splitting the sample in two, between subjects with values of methylation falling above or below the median value.

## Results

### Methylation analysis of rs6265 BDNF polymorphism

Methylation analysis was focused upon genomic region including one CpG site that can be created or abolished by rs6265 SNP (Fig 1A). Before methylation analysis, we performed genotyping for this polymorphism in our cohort of study using HRM technique [27].

We identified 122 subjects (58.4% of total sample) carrying the Val/Val genotype, 81 subjects (38.8%) with Val/Met genotype, and 6 patients (2.8%) carrying the Met/Met genotype.

Since the group of patients homozygous for the Met allele was very small, we conducted further analysis considering subjects carrying (Met+) or not carrying (Met-) the Met allele.

By qRT-PCR we found that the mean methylation percentage was 80.9 (SD = 16.96; median = 81). Methylation percentage of subjects carrying the Val/Val (mean = 74.58; SD = 10.821), Val/Met (mean = 94.53; SD = 10.722) and Met/Met (mean = 28.50; SD = 2.665) genotypes was significantly different (F_2,206_ = 160.19; p<0.001) (Fig 1B). These result showed the evidence for an association between rs6265 SNP and DNA methylation in MS patients.

### Effect of the rs6265 SNP and BDNF methylation on disease progression: univariate analysis

During the period of interest, 52 failures (i.e., reaching an EDSS score of 6) were reported.

Table 1 reports the incidence rates of the failure. As shown, the failure rate was evenly distributed between the patients carrying or not carrying the Met allele (exposed = Met+; IRD = -0.009; 95% confidence interval [95%CI] = -0.0200–0.0014; p = 0.102). When the sample was stratified according to the percentage of the BDNF methylation, subjects falling below the median (median methylation = 81%) were at higher risk of failure (IRD = 0.016; 95%CI = 0.0050–0.0279; p = 0.004).

**Table 1:** Incidence rates and incidence rate differences (IRD) of subjects stratified according to BDNF gene polymorphism and BDNF gene methylation. The exposed are respectively the patients carrying the Met Allele and those presenting a methylation level below 81% while the unexposed are respectively the patients not carrying the Met allele and presenting a methylation level above 81%. 95%CI: 95% confidence interval.

	Exposed			Unexposed			IRD	95%CI		p
	Failures	Time at risk (years)	Incidence rate (event/person/year)	Failures	Time at risk (years)	Incidence rate (event/person/year)				
**BDNF polymorphism**										
Val/Val	36	1496	0.024							
Val/Met	12	1005	0.012							
Met/Met	4	76	0.053							
Met+	16	1081	0.015	36	1496	0.024	-0.009	-0.0200	0.0014	0.102
**BDNF gene methylation**										
<81%	34	1165	0.029	18	1412	0·013	0.016	0.0050	0.0279	0.004

**Fig 2:**
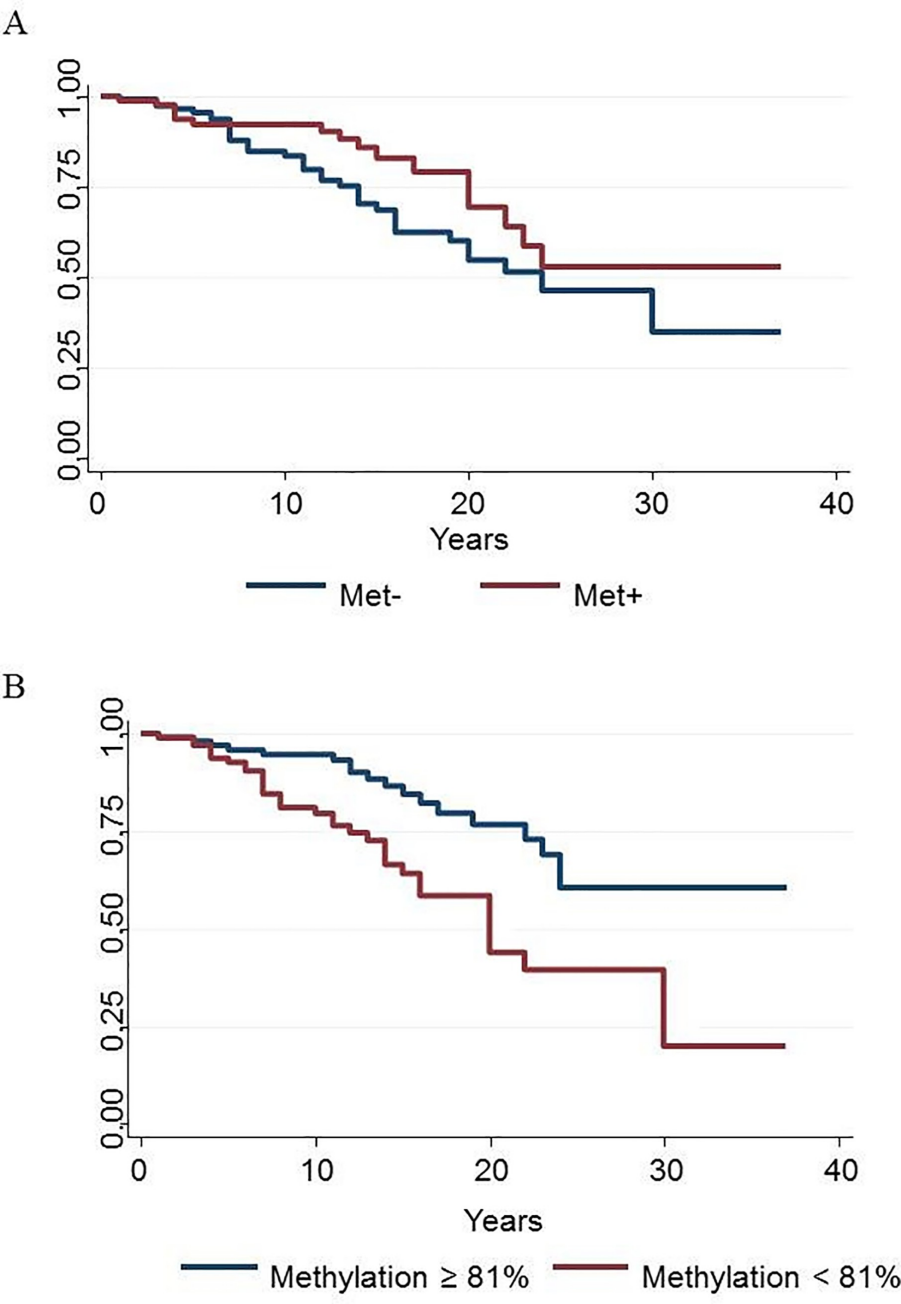
Survival estimates according to rs6265 SNP and *BDNF* percentage of methylation. A) survival estimates of subjects carrying (Met+) or not carrying (Met-) the Met allele of the rs6265 SNP. B) survival estimates of subjects with *BDNF* methylation above or below the median (81%).

Fig 2 displays the survival estimates of the sample stratified according to rs6265 SNP and *BDNF* percentage of methylation, respectively.

### Multiple variables Cox’s regression analysis

Table 2 displays the results of the multiple variables regression analysis, in which several demographic (age, gender) and disease-related variables (clinical relapses; MRI relapses; ARR; MRI-ARR; treatment) were entered alongside with rs6265 SNP and *BDNF* methylation.

**Table 2:** Results of the multiple variables Cox’s regression analysis. ARR: annualized relapse rate; ARR-MRI: annualized MRI relapse rate; HR: hazard ratio; SE: standard error; 95%CI: 95% confidence interval.

	HR	SE	z	p	95%CI	
Age	1.04	0.017	2.30	0.022	1.006	1.073
Gender (F)	0.56	0.200	-1.62	0.105	0.277	1.128
Clinical relapses	1.14	0.283	0.53	0.596	0.701	1.854
ARR	2.03	0.965	1.49	0.135	0.801	5.154
MRI relapses	0.80	0.472	-0.38	0.701	0.250	2.542
ARR-MRI	0.89	0.535	-0.20	0.843	0.272	2.896
First line treatment	1.37	0.592	0.73	0.463	0.589	3.195
Second line treatment	1.56	1.271	0.54	0.589	0.314	7.716
Third line treatment	3.60	2.170	2.12	0.034	1.104	11.731
BDNF gene polymorphism (Met+)	0.74	0.439	-0.51	0.613	0.232	2.366
BDNF gene methylation (<81%)	2.72	1.382	1.97	0.049	1.005	7.365
Polymorphism X Methylation	2.36	1.890	1.07	0.283	0.491	11.339

As shown, the occurrence of the failure (EDSS = 6) was predicted by age (Hazard Ratio [HR] = 1.04; 95%CI = 1.006–1.073; p = 0.022) and by a percentage of methylation below 81% (HR = 2.72; 95%CI = 1.005–11.339; p = 0.049).

Finally, subjects with more aggressive disease, treated with third line agents, were at higher risk of reaching the outcome (HR = 2.17; 95%CI = 1.104–11.731; p = 0.034).

## Discussion

Several studies have tried to correlate the role of the most common rs6265 BDNF polymorphism with the prognosis of patients affected by MS, with contradictory data so that other mechanisms are thought to be involved in the modulation of *BDNF* gene [20,21].

Recent advances in the field of epigenetics, extended the role of epigenetic mechanisms, as methylation, in controlling key biological processes. Our study shows that the rs6265 SNP taken by itself does not link to a different chance of reaching a more severe EDSS score. On the contrary, a lower percentage of methylation of the *BDNF* gene, regardless of its polymorphism, links to higher odds in reaching an important disability. Considering a higher methylation as a “silencer” of the gene, this result can be translated affirming that a lower inhibition of the gene links to higher odds in reaching an EDSS score of 6.0.

Considering the BDNF as a neurotrophic factor we can assume the percentage of methylation of the *BDNF* gene as a consequence of the disease activity. Patients with a more severe inflammation could appeal to a de-methylation, hence to a higher secretion of BDNF, in order to preserve the functions of the CNS. The same patients could be the one that, by exploiting at a faster rate the functional reserve of the brain, tend to reach a more severe disability score. On the contrary, patients with a mild or moderate disease activity, could keep the *BDNF* gene hyper-methylated and tend to maintain a lower EDSS score.

Our study strongly suggests that the *BDNF* methylation, considered as an epiphenomenon of the disease activity, might help to discriminate patients with a higher inflammatory process from patients with a lower degree of inflammation. If confirmed by further studies, this could become a first reliable prognostic factor in MS potentially helpful for clinicians to distinguish patients with a more severe disease from those with a milder one. Therefore, the use of a such prognostic factor in MS could greatly help the neurologist to identify the patients that could benefit from a more aggressive and earlier therapeutic approach or of an induction strategy as more appropriate treatment.

A better understanding of the *BDNF* machinery and of its epigenetic regulatory mechanism could impact the management of MS patients facilitating selection of a patient-tailored therapy, thus greatly improving the quality of life of patients and also the comprehension of the disease pathophysiology.

## Supporting information

S1 Table Study dataset. Dataset with the polymorphism and the methylation levels of the BDNF gene for each patient.
(XLSX)
