# Correction: BDNF rs6265 polymorphism methylation in Multiple Sclerosis: A possible marker of disease progression

**DOI:** 10.1371/journal.pone.0212906

**Published:** 2019-02-21

**Authors:** Viviana Nociti, Massimo Santoro, Davide Quaranta, Francesco Antonio Losavio, Chiara De Fino, Rocco Giordano, Nicole Piera Palomba, Paolo Maria Rossini, Franca Rosa Guerini, Mario Clerici, Domenico Caputo, Massimiliano Mirabella

The seventh author’s name is incorrect. The correct name is: Nicole Piera Palomba.
